# Investigation on the Microstructure and Mechanical Properties of CNTs-AlSi10Mg Composites Fabricated by Selective Laser Melting

**DOI:** 10.3390/ma14040838

**Published:** 2021-02-09

**Authors:** Shixuan Luo, Ruifeng Li, Peiyuan He, Hangyu Yue, Jiayang Gu

**Affiliations:** 1 School of Materials Science and Engineering, Jiangsu University of Science and Technology, Zhenjiang 212003, China; zmluoshixuan@gmail.com (S.L.); yh17368088912@163.com (P.H.); yuehangyuyhy@just.edu.cn (H.Y.); 2 Marine Equipment and Technology Institute, Jiangsu University of Science and Technology, Zhenjiang 212003, China; gujiayang@126.com

**Keywords:** CNT-AlSi10Mg, selective laser melting, microstructure, properties

## Abstract

CNT-AlSi10Mg composites fabricated by SLM have drawn a lot attention in structural application due to its excellent strength, elasticity and thermal conductivities. A planetary ball milling method was used to prepare the carbon nanotube (CNT)-AlSi10Mg powders, and the CNT-AlSi10Mg composites were fabricated by selective laser melting (SLM). The density, microstructure and mechanical properties of CNT-AlSi10Mg composites were studied. The density of the test samples increased at first and then decreased with increasing scan speed. When the laser scan speed was 800 mm/s, the test sample exhibited the highest density. The hardness increased by approximately 26%, and the tensile strength increased by approximately 13% compared to those values exhibited by the unreinforced AlSi10Mg. The grains of CNT-AlSi10Mg composite are finer than that in the AlSi10Mg. The CNTs were distributed along the grain boundaries of AlSi10Mg. Some of the CNTs reacted with Al element and transformed into Al_4_C_3_ during SLM, while some of the CNTs still maintained their tubular structure. The combination of CNTs and Al_4_C_3_ has a significant improvement in mechanical properties of the composites through fine grain strengthening, second phase strengthening, and load transfer strengthening.

## 1. Introduction

Metal matrix composites (MMCs) are composed of two or more materials and customized performance can be obtained. Particle-reinforced MMCs have attracted plenty of attentions due to their excellent mechanical properties [1]. Among those MMCs produced in recent years, aluminum matrix composites (AMCs) have received much attention. Lots of AMCs have been investigated with different phases: Al_2_O_3_ nanoparticles [2], SiC nanoparticles [1], TiC nanoparticles [3], and TiB_2_ nanoparticles [4].

CNTs have become an important class of materials in engineering and functional equipment industry due to their excellent performance, and electrical and thermal conductivity. CNT-AMCs have been prepared through a variety of processes. In the powder metallurgy method, AMCs and CNTs are mixed by grinding, followed by consolidation through hot static pressure or spark plasma sintering [5,6,7,8]. The limitation of this method is that the preparation of the powders is quite complicated. CNTs reinforced AMCs can be produced by friction stir process [9,10]. Although many processing techniques have been reported on preparing CNT-AMCs, but it still remains an ongoing challenge due to strict requirements for the manufactured process.

Selective laser melting (SLM) is a laser additive manufacturing technology (AM), which uses laser beam to melt metal powders along a given path quickly and completely. Because of its rapid prototyping ability, SLM is widely used in the production of various metals and alloys [11,12,13,14]. It has the following advantages: capable of producing complex-shaped three-dimensional parts [15], reducing manufactured time and cost [16], flexible alloy design [17], and superior mechanical properties [18]. High-energy laser irradiation in the SLM process can produce a stronger metallurgical bond between the alloy matrix material and the reinforcement material in MMCs [19]. Recently, there was a lot of studies on the use of SLM to manufacture MMCs, compared with other conventional processing methods, the properties of the MMCs have been greatly improved. Wen et al. [20] studied the mechanical properties of RGO (reduced graphene oxide)-S136 composites fabricated by SLM and found that the tensile strength of these parts reached 535.3 MPa; Song et al. [21] reported that the tensile strength of Fe-SiC composites fabricated by SLM was 753 ± 49 MPa, which was much higher than pure iron (357 ± 22 MPa). Zhao et al. [22] fabricated SiC-AlSi10Mg composites via SLM, and found that the hardness of these composites was much higher than that of as-cast AlSi10Mg alloy. Li et al. [23] investigated the mechanical properties of nano-TiB_2_ decorated AlSi10Mg (NTD-Al) composites fabricated by SLM, and the results showed a high tensile strength of 530 MPa, excellent ductility of 15.5% and microhardness of 191 HV0.3, which were higher than most of the wrought and tempered Al alloys. However, according to the above review of previous studies, CNTs reinforced MMCs produced by SLM are rarely reported.

Recently, there have been lots of researches on the CNT-AlSi10Mg composites. However, these conventional processes would weaken the effect of CNTs due to long processing time, it is difficult to fabricate composites with complex geometries. In the present work, the CNT-AlSi10Mg powder was prepared through planetary ball milling process, and CNT-AlSi10Mg samples were fabricated by SLM. The microstructure and mechanical properties of CNT-AlSi10Mg composites were studied.

## 2. Experimental

### 2.1. Material and Procedure

**Figure 1:**
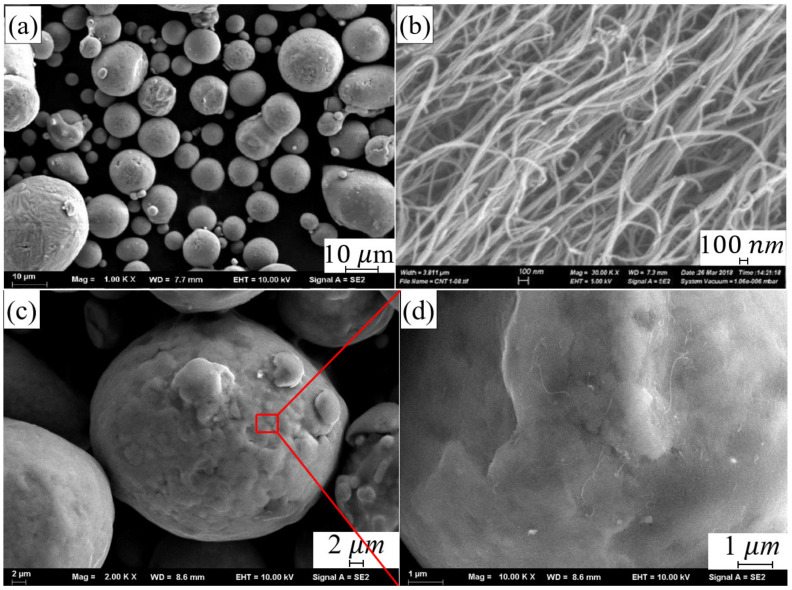
Micro-morphology of (**a**) AlSi10Mg powder, (**b**) MWCNTs, (**c**) CNT-AlSi10Mg powder and (**d**) Schematic diagram of CNT attached to AlSi10Mg powder.

The AlSi10Mg powder was supplied by SLM Solution Group AG Co., Ltd. (Lubeck, Germany). The chemical composition is shown in Table 1. The particle size of the powder is between 10 μm and 50 μm (as shown in Figure 1a).

Multi-walled CNTs (MWCNTs) was supplied by Chengdu Organic Chemicals Co., Ltd., Chengdu, China, which exhibited a diameter of about 8–15 nm and a length of less than 20 μm, as shown in Figure 1b. The composite powder was prepared through planetary ball milling process. The CNTs with a mass fraction of 1% and the AlSi10Mg with a mass fraction of 99% were mixed by a planetary ball milling process with a ball-to-powder weight ratio of 2:1. The rotation speed was 300 rpm and total milling time was 4 h. Figure 1c,d shows the micro-morphology of composite powder after ball milling.

The composite was fabricated using NCL-M2120 SLM machine (maximum output of 400 W, λ = 1064 μm, a spot size of 0.07 mm). The fabrication of the composite was performed in the argon atmosphere to prevent oxidation. Samples (8 × 8 × 5 mm^3^) was fabricated by different scan speeds 400, 600, 800, 1000, and 1200 mm/s. The hatch space was 70 μm and the laser power was 160 W. The layer thickness was 20 μm and the angle of scan direction between neighboring layers was 67°.

### 2.2. Characterization

All test samples were cut from the substrate via EDM (electrical discharge machining, BM360, SUZHOU BAOMA, Suzhou, China). Metallographic testing specimens were prepared according to the standard procedures. Samples were etched with Keller reagent (2.5 mL HNO_3_, 1.5 mL HCL, 1 mL HF, 95 mL H_2_O) for 12 s and observed by OM. A JSM-6480 field emission scanning electron microscope (JEOL) was used to observe the composite powder morphology and microstructure of the samples. XRD characterizations were conducted on an XRD-6000 (Shimadzu, Kyoto, Japan) using a Cu target, 40 KV voltage in the angular range 20°–90°. TEM analysis was characterized on JEM-2100F (JEOL, Tokyo, Japan). The porosity was calculated by the Image J software. The Vickers hardness was measured using an HXS-1000AC hardness tester (Shanghai Shangguang Optical Co., Ltd. Shanghai, China) at a load of 100 g and an indentation time of 15 s. Tensile strength was tested using GB/T2651-2008 standard with a strain rate of 1 mm/min.

## 3. Results and Discussion

### 3.1. The Effect of Scan Speeds on Density

**Figure 2:**
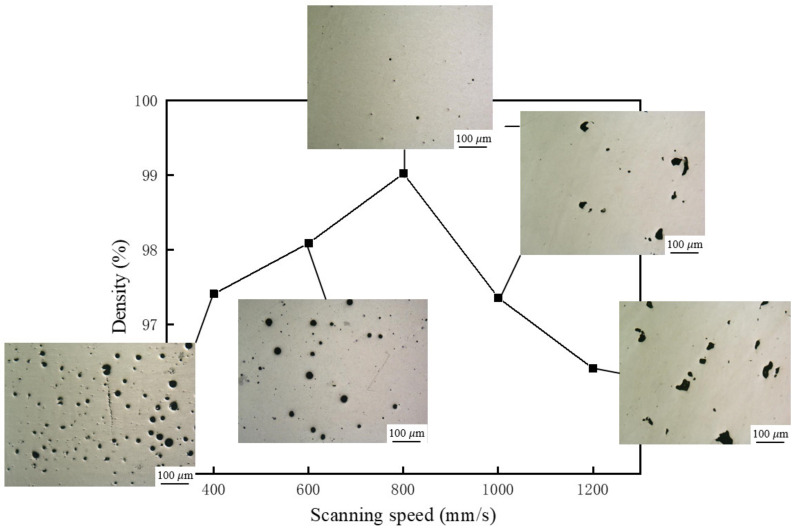
Variation of density and micro-pores at different scanning speeds.

Figure 2 shows the effect of scan speed on the density of the samples. It can be found that the density of the samples increased at first, and then decreased with increasing scan speed. When the laser scan speed was 800 mm/s, the test samples exhibited the highest density of 99%. To investigate the effect of scan speed on the density of the samples, un-etched samples were additionally polished and the metallographic pictures of the sample at different scanning speeds were obtained. The pores inside the samples are divided into two types. One is the regular spherical pores with a volume of less than 100 μm, which are metallurgical pores. The other one is irregular pores and the volume fraction is larger than 100 μm, which is lack of fusion. It can be found that metallurgical pores are formed at a relatively slow scanning speed, and lack of fusion pores are formed at a relatively faster scanning speed.

When the scan speed is lower than 800 mm/s, there are two reasons for the formation of metallurgical pores. One reason is that the melt pool temperature is high and the existing time is long due to the low scan speed. At this temperature, the solubility of the shielding gas in the liquid metal is high. Due to the fast cooling rate of SLM, the gas cannot escape from the molten pool in time and is trapped in the molten pool. Therefore, a lot of metallurgical pores were formed inside the sample. Another reason is that there are some low-melting elements such as aluminum and magnesium in the composite powder. When the heat input is large, these elements will be evaporated and the gas flowing inside the molten pool has a violent interaction, which will form recoil pressure on the molten pool. The recoil pressure will cause liquid phase in the molten pool to splash. The recoil pressure on the molten pool and the evaporation behavior of the liquid phase will form small “keyholes” inside the molten pool, which form metallurgical porosity defects after solidification.

When the scan speed exceeds 800 mm/s, the heat input is small, and the temperature of the molten pool is low. The solubility of gas in liquid metal is low, and the recoil pressure of the metal vapor to the molten pool is reduced. Therefore, it is difficult to form “keyholes” inside the molten pool. When the scan speed increased, the lack of fusion was formed inside the samples. Due to the low heat input at high scan speed, the melting tracks were smaller and unstable. Unmelted powders remained in the melting path when the inter-pass overlap is not properly maintained. The unmelted powders are difficult to re-melt completely when a new layer is deposited, which leads to the formation of lack of fusion defects in the samples.

### 3.2. Microstructure

**Figure 3:**
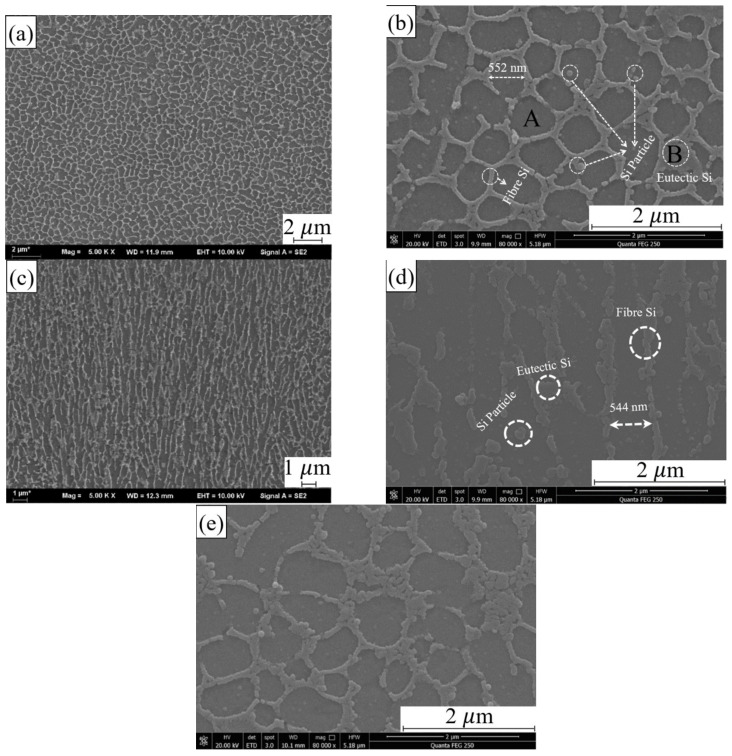
The microstructure of SLMed CNT-AlSi10Mg (**a**) in XY plane and (**b**) XY plane in high magnification. The microstructure of SLMed CNT-AlSi10Mg microstructure in (**c**) XZ plane and (**d**) XZ plane in high magnification. (**e**) The microstructure of SLMed AlSi10Mg.

Figure 3a,b shows the microstructure of SLMed CNT-AlSi10Mg in the XY plane, it can be found that two phases in the composite. The EDS results of two regions are shown in Table 2. The main component of region A is Al with 90.42%. The content of Si in region B is 23.59%, which greatly exceeds the solubility of Si in the solution. Therefore, it is considered that the main component of region B is eutectic Si, which is similar to the results of [24]. The morphology of eutectic Si was fibrous, and the eutectic Si distributed in the continuous network. The grey islands are α-Al phases. The α-Al solidifies first leaving residual Si at the grain boundaries [25]. The solid solution of Si in Al is easily decomposed during the slow cooling process. Elemental Si precipitated in the form of relatively coarse particles to obtain a continuous eutectic structure of Al and Si, and dispersed α-Al phase was obtained. Therefore, a unique microstructure is formed in the sample due to rapid melting and subsequent rapid cooling during SLM process.

Moreover, it can be seen from Figure 3a,b, the brighter parts in the figure are the eutectic Si phases, precipitated from the Al matrix. The darker parts are the α-Al phases. The α-Al phase is divided into equiaxed islands by Si phase, which makes the α-Al in the form of cellular equiaxed grains with average diameter of 593 nm. It can be seen from the small white circle in Figure 3b that the Si phase presents three morphologies in the Al matrix, one is fibrous, and its diameter is about 72–89 nm. Another one gathered in a spot is eutectic Si. The last one is granular with a diameter of about 33–56 nm, which are distributed on the α-Al phase or connected to the fibrous Si phase.

The microstructure on the XZ plane (Figure 3c,d) is different from the cellular equiaxed grains exhibited by the α-Al phase on the XY plane. The α-Al phase is elongated and shows a dendrite structure parallel to building direction, and the width is about 544 nm. The fibrous Si phase precipitated from the Al matrix is still gathered at the grain boundary, and it can be seen that the Si phase exhibits two morphologies in the Al matrix, one is fibrous and its diameter is about 72–89 nm, and the other is in the form of granular distributed on the α-Al phase with a diameter of about 100 nm.

Figure 3e shows the microstructure of SLMed AlSi10Mg, the grain size in the AlSi10Mg is about 0.84 μm, while the grain size in the CNT-AlSi10Mg is about 0.58 μm, as shown in Figure 3b. Therefore, the addition of CNTs into AlSi10Mg will make the crystal grains finer. This result was similar with the result of [26], which showed the grain size of Al alloy is reduced from 5 μm to 1 μm by adding 2.0 wt% CNTs. The CNTs distribute along the grain boundaries of the grains and still maintain their tubular structure. The CNTs located at the grain boundaries effectively hinder the grain growth by the pinning effect and make the grains finer.

**Figure 4:**
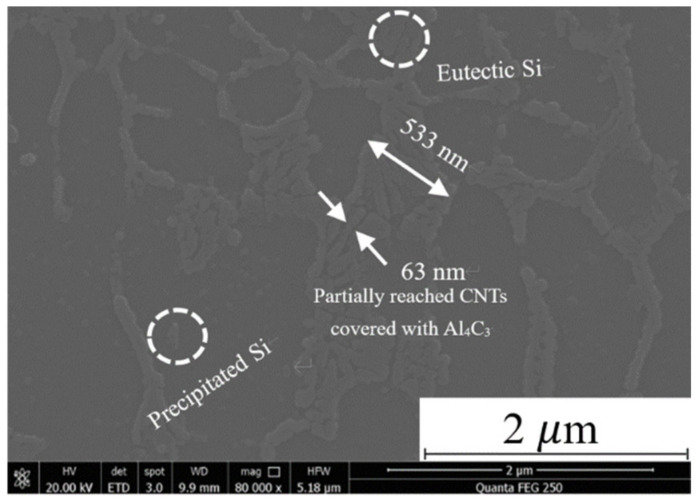
Microstructure of CNT-AlSi10Mg specimen after laser selection melting.

It can be found that in addition to the eutectic Si aggregation at the grain boundary of the α-Al phase, it is also found a nano-scale tubular structure with a length of 533 nm and a width of 63 nm at the grain boundary in the CNT-AlSi10Mg sample, as shown in Figure 4. EDS analysis of this structure is shown in Table 3. The element C was found besides Al, Si and Mg elements that are commonly found in AlSi10Mg, which indicated CNTs and the in situ derivative phases were produced by the CNTs and the matrix in the structure. It is believed to be CNTs covered with Al_4_C_3_ on the outer wall.

**Figure 5:**
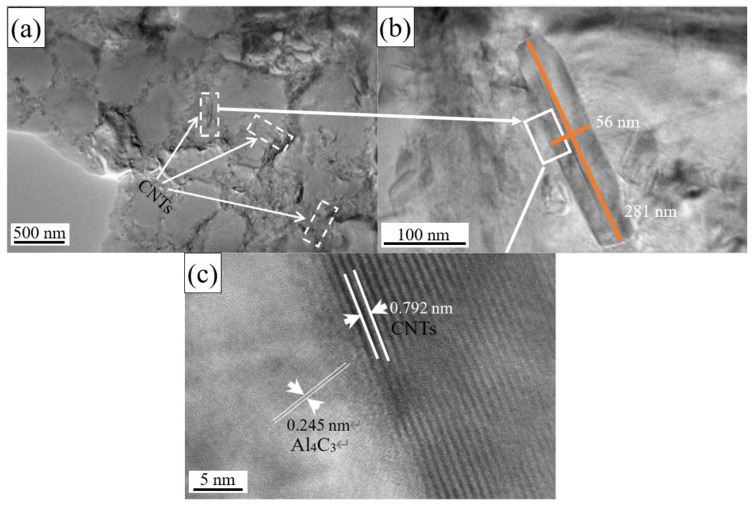
(**a**,**b**) TEM analysis of CNTs in the test samples. (**c**) The HRTEM of CNTs in the test samples.

To confirm that the structure is tubular CNTs covered with Al_4_C_3_ on the outer wall, TEM analysis was conducted and the results are shown in Figure 5. The CNTs still maintain the original tubular structure distributed along the grain boundaries with a length of approximately 300–400 nm and a width of 40–50 nm (see Figure 5a). Compared with the original CNTs with a length of about 20 μm, the length of CNTs is greatly reduced after the SLM, but the aspect ratio is still between 6–8, which makes the CNTs maintain a certain load capacity [27]. The CNTs located at the grain boundaries effectively hinder the movement of dislocations by the pinning effect and improve the mechanical properties.

Figure 5c shows the HRTEM of CNTs in the samples, and the layered carbon structure can be seen at the interface between Al matrix and the CNTs. The interlayer spacing between the CNTs walls is 0.792 nm. The left side of the interface is the Al_4_C_3_ matrix with a lattice parameter of 0.245 nm. Long and entangled CNTs will be divided into several small clusters during the process of preparing the CNT-AlSi10Mg composite powder with planetary ball milling process. These small clusters of CNTs will be dispersed on the surface of the particles in the AlSi10Mg powder. CNTs cannot be used as a nucleation site for Al [28], so the CNTs is squeezed to the grain boundaries and maintained its tubular structure. However, CNTs will have many crystallographic defects during the ball milling process, which will reduce the stability of CNTs at a high temperature [29]. SLM process usually involves high temperature, the laser energy is first absorbed by CNTs, part of the CNTs are decomposed into carbon atoms in the molten pool. As the temperature in the molten pool is high, the Marangoni flow increases and leads to the vibration of the melt, which promotes the diffusion of carbon atoms and thus increases the nucleation rate of Al_4_C_3_ [29]. The in situ reaction occurred at the interface between CNTs and the Al matrix, and the Al_4_C_3_ was formed on the outer layer of the CNTs.

**Figure 6:**
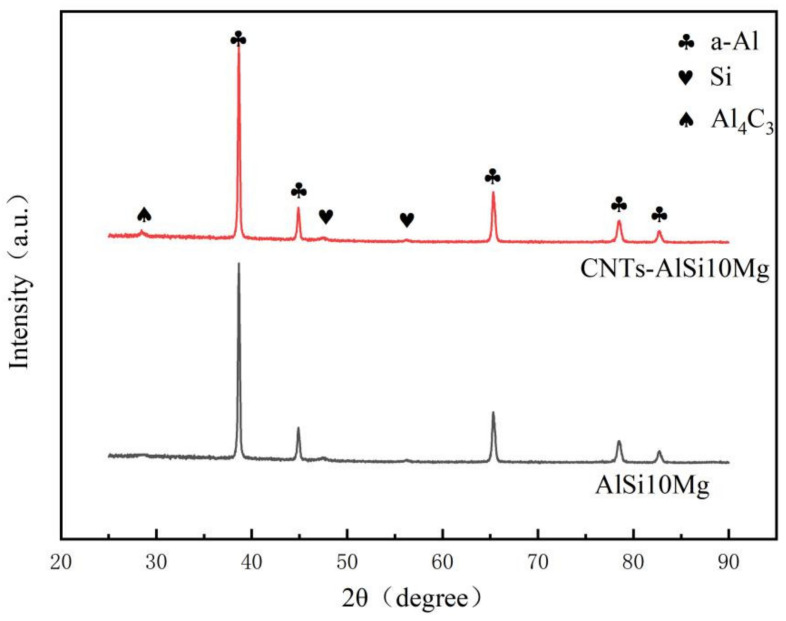
X-ray diffraction patterns of SLMed CNT-AlSi10Mg and AlSi10Mg.

Figure 6 illustrates X-ray diffraction patterns of SLMed CNT-AlSi10Mg and AlSi10Mg. It can be found that only the α-Al phase and the Si phase were detected in the SLMed AlSi10Mg. The α-Al phase has different diffraction peaks, indicating that the α-Al phase has different orientations. The diffraction intensity of α-Al phase is greater than that of Si phase. In addition to the α-Al phase and Si phase, the Al_4_C_3_ was detected due to the effect of the high temperature in the SLM process. The ball-milled CNTs will be decomposed into carbon atoms. The carbon atoms and Al atoms combined and reacted to form Al_4_C_3_ phase, and increase the interface bonding between CNTs and Al matrix.

The Gibbs free energy function method is used to determine whether there will be spontaneous reactions in the process to form possible phases, which is defined as:

(1) $$\Phi_{T}=-\frac{G_{T}^{0}-H_{T_{0}}^{0}}{T}$$

where $\Phi_{T}$ is the Gibbs free energy function of the substance, $G_{T}^{0}$ is the standard free energy of the reaction substance at temperature T, $H_{T_{0}}^{0}$ is the free enthalpy value of the reaction substance at a certain reaction temperature $T_{0}$.

According to the theory of thermodynamics, both free energy G and free enthalpy H are state functions. Secondly, at the phase transition temperature, the free energy G function and free enthalpy H function are still continuous, so the material Gibbs free energy function $\Phi_{T}$ is also a state function. Thus, Equation (1) can be changed to:

(2) $$\Delta \Phi_{T}=\Delta \left(-\frac{G_{T}^{0}-H_{T_{0}}^{0}}{T}\right)=-\frac{\Delta G_{T}^{0}-\Delta H_{T_{0}}^{0}}{T}$$

The change value of Gibbs free energy function ${\Delta \Phi }_{T}$ can be obtained by the value of Gibbs free energy function $\Phi_{T}$:

(3) $$\Delta \Phi_{T}=\sum {\left(n_{i}\Phi_{i,T}\right)}_{\mathrm{resultant}}-\sum {\left(n_{i}\Phi_{i,T}\right)}_{\mathrm{reactant}}$$

The following free energy change formula can be derived from Equation (2):

(4) $$\Delta G_{T}^{0}=\Delta H_{T_{0}}^{0}-T\Delta \Phi_{T}$$

Similarly, according to the theory of thermodynamics, the thermochemical constant with a pure substance temperature T_0_ of 298 K is generally taken, so according to Equations (2) and (4):

(5) $$\Delta G_{T}^{0}=\Delta H_{T_{0}}^{0}-T\Delta \Phi_{T}^{\prime }$$

(6) $$\Phi_{T}^{\prime }=-\frac{G_{T}^{0}-H_{298}^{0}}{T}$$

The results of the Gibbs free energy value calculated according to the temperature of each substance are written in the form of $\Delta G_{T}^{0}=A+BT$ using regression analysis. This study mainly used AlSi10Mg as the matrix powder to study the strengthening effect of carbon nanotubes on CNT-AlSi10Mg composites. Considering the corresponding elements and compounds, there are a variety of reaction systems to choose from. The main reaction systems and the chemical reactions that may be involved are:

4Al + 3C = Al_4_C_3_ (7)

2Mg + Si = Mg_2_Si (8)

Using regression analysis method, change the above calculation result to the form of $\Delta G_{T}^{0}=A+BT$:

4Al + 3C = Al_4_C_3_; ΔG^0^_T_ = −216,689.64 + 143.88T (9)

2Mg + Si = Mg_2_Si; ΔG^0^_T_ = −79,180.31 + 16.34T (10)

**Figure 7:**
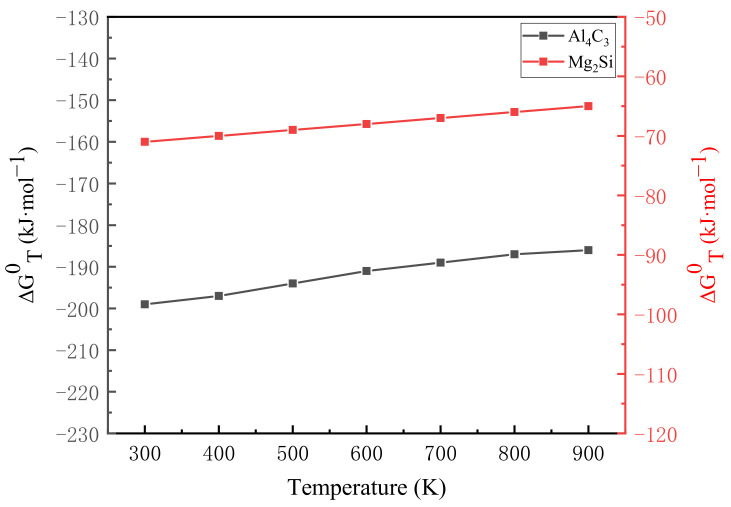
Relation between the Gibbs free energy and temperature T.

According to the calculation results of Equations (9) and (10), the relationship between the Gibbs free energy $\Delta G_{T}^{0}$ of each reaction and the temperature T is drawn, as shown in Figure 7. It can be seen that when the temperature is between 298 K and 922 K, the Gibbs free energy $\Delta G_{T}^{0}$ has a linear relationship with the temperature T, and $\Delta G_{T}^{0}$ changes with the temperature. The value of $\Delta G_{T}^{0}$ reflects the reaction trend. The smaller the value of $\Delta G_{T}^{0}$, the greater the trend of the reaction, the greater the driving force for the formation of reaction products, and the more stable the formed products. It can be seen from Figure 7 that the value of Gibbs free energy $\Delta G_{T}^{0}$ is negative when the reactions of Equations (7) and (8) are carried out. However, the Gibbs free energy $\Delta G_{T}^{0}$ when Al_4_C_3_ is formed is much smaller than that when Mg_2_Si is formed., that is, Al_4_C_3_ has the strongest stability in the reaction process, and it is also formed first in other earlier reactions. Mg_2_Si has a relatively large Gibbs free energy $\Delta G_{T}^{0}$ and is not formed in the reaction. The previous XRD analysis was further verified, and it was proved that Al_4_C_3_ was formed during the laser melting of CNT-AlSi10Mg.

### 3.3. Mechanical Properties

**Figure 8:**
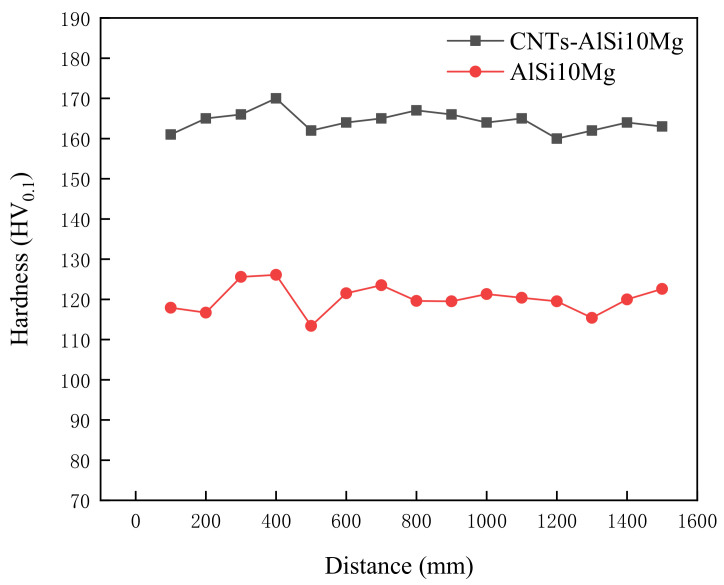
Microhardness of SLMed CNT-AlSi10Mg and AlSi10Mg.

Figure 8 shows the microhardness of SLMed CNT-AlSi10Mg and AlSi10Mg. It can be found that the maximum microhardness of the CNT-AlSi10Mg is about 151.17 HV0.1, and the microhardness of the AlSi10Mg is about 120.15 HV0.1. Compared with the AlSi10Mg alloy, the microhardness of the composite was greatly increased by about 26% after adding CNTs to the AlSi10Mg powder. The reason was that the grains of CNT-AlSi10Mg are refined by CNTs. During the laser selective melting process, the cooling speed is fast, which limits the diffusion of alloying elements and the growth of grains, so that nano-scale fibrous eutectic Si and cellular equiaxed α-Al can be obtained which play the role of fine-grain strengthening, and the existing of the molten pool is short, the Si phase in the alloy has no time to precipitate out, and solid-dissolved in the Al matrix, which plays the role of solid-solution strengthening. The dual effects of solid solution strengthening and fine grain strengthening make the microhardness after laser selective melting is much higher. At the same time, since the carbon nanotubes are added to the AlSi10Mg powder, the outer wall of the added carbon nanotubes reacts in situ with the matrix to form Al_4_C_3_ in-situ interface compounds. In addition, compared with parts without adding CNTs, melting and solidification in the SLM process strengthens CNTs, making them as nucleation site, which can increase microhardness [30]. The stable interface helps to load between the matrix and the reinforcing phase, increasing the hardness of the material.

**Figure 9:**
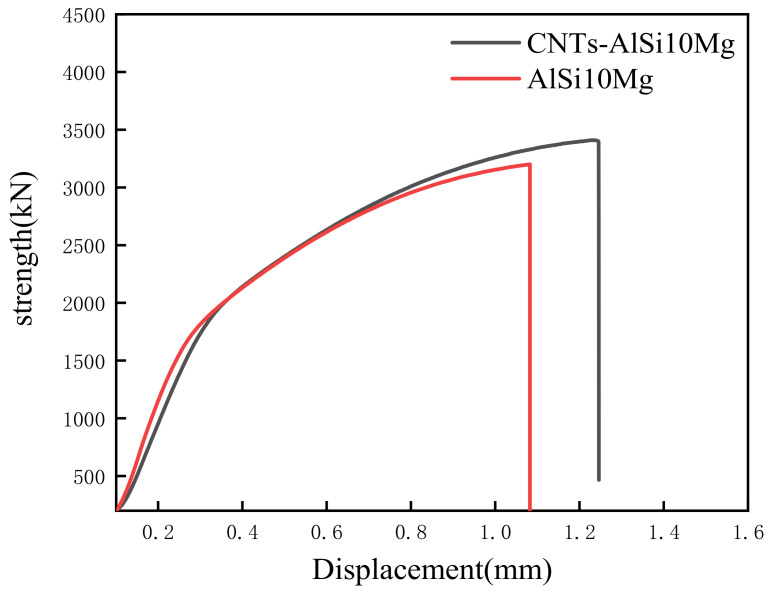
Strength-displacement curve of tensile specimen at room temperature.

**Figure 10:**
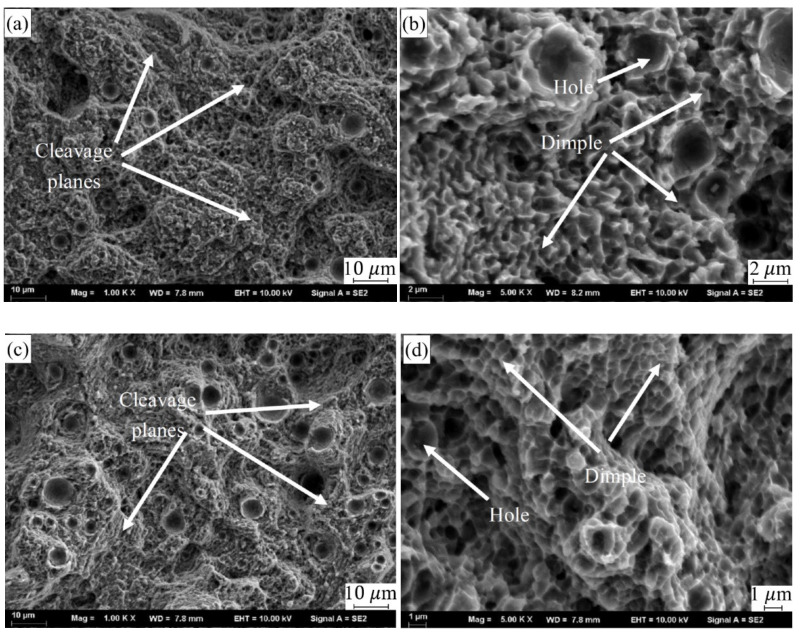
SEM of fracture morphology: (**a**) fracture morphology of CNT-AlSi10Mg, (**b**) high magnification of (**a**,**c**) fracture morphology of AlSi10Mg, and (**d**) high magnification of (**c**).

Figure 9 illustrates the force-displacement curve of SLMed CNT-AlSi10Mg and AlSi10Mg at room temperature. Table 4 shows the tensile properties of samples at room temperature. CNTs distributed in the matrix will form a strong interfacial bonding which helps in load transfer when under deformation and improve tensile strength [31]. The average tensile strength, yield strength, and elongation of SLMed AlSi10Mg are 439.2 MPa, 270.7 MPa, and 7.5% respectively. The average tensile strength, yield strength, and elongation of SLMed CNT-AlSi10Mg composite are 498.6 MPa, 309.6 MPa, and 10.6% respectively. Compared with AlSi10Mg, the tensile strength of the CNT-AlSi10Mg was increased by 13.5%, and the yield strength was increased by 12%. To investigate the fracture mechanism of the composite, fracture morphology was analyzed, as shown in Figure 10. It can be found in Figure 10a,c that there are cleavage steps at the fracture position in both samples, which is characterized by intergranular fracture. High magnification SEM of fracture morphology is shown in Figure 10b,d, it can be found that there are some dimples with a size of 1–2 μm on the cleavage planes, and the dimples are regular in shape but different in depth. The deep dimples indicate high elongation in the SLMed CNT-AlSi10Mg, while the shallow dimples indicate low elongation in the SLMed AlSi10Mg.

As can be found from the above experimental results, SLMed CNT-AlSi10Mg existed three strengthening mechanisms. The first one is fine grain strengthening, and the grains become finer after adding CNTs into AlSi10Mg. The grain refinement can form more grain boundaries, which will form dislocation accumulation and hinder the movement of dislocation. Therefore, grain refinement improves the mechanical properties. The second one is second phase strengthening. Nano-scale eutectic Si precipitated in the α-Al phase, and the CNTs outer wall covered with Al_4_C_3_ at the grain boundary, which strengthens the Al matrix. During plastic deformation, the nano-scale structures hinder the movement of dislocations and resulted in dislocations bent between nano-structures. Dislocation bending generates back stress, which prevents further migration of dislocations and increases the strength of the composite. The third one is load transfer strengthening, the CNTs outer wall covered with Al_4_C_3_ are formed in CNT-AlSi10Mg, which improves the stability of the interface. During the loading process, the load can be effectively transferred from the substrate to CNTs.

## 4. Conclusions

CNT-AlSi10Mg powders were prepared by the planetary ball milling method, and CNT-AlSi10Mg composites were fabricated by SLM. The microstructure and mechanical properties of SLMed CNT-AlSi10Mg were studied as well as the density and phase composition. Conclusions can be drawn as follows:

1. The density of the composites increased at first then decreased with increasing scan speed. A laser scan speed of 800 mm/s produced the highest density: 99%. When the scan speed is relatively slow, regular round metallurgical porosities are formed in the composites, and when the scan speed is relatively fast, irregular lack of fusion defects are formed.
2. Al_4_C_3_-CNTs with a length of 533 nm and a width of 63 nm were found at the grain boundaries of the α-Al and exhibited the original tubular structure.
3. The hardness and tensile strength of SLMed CNT-AlSi10Mg increased by 26% and 13%, respectively, compared to those of the unreinforced AlSi10Mg.
4. SLMed CNT-AlSi10Mg has three strengthening mechanisms: fine grain strengthening, second phase strengthening, and load transfer strengthening.

## Figures and Tables

**Table 1:** AlSi10Mg powder chemical composition (wt%).

Elements	Si	Fe	Mg	Mn	Zn	Ti	Cu	Al
contents	10.13	0.07	0.10	0.06	0.026	0.02	0.03	Bal.

**Table 2:** EDS results of region (wt%).

Elements	Al	Si	Mg	O	C
A	90.42	7.99	0.38	1.21	0
B	65.28	23.59	0.68	1.66	8.79

**Table 3:** EDS results of nanotube structures (wt%).

Elements	Al	Si	Mg	C	O
wt%	73.34	10.04	0.42	13.78	2.42

**Table 4:** Tensile properties of specimen at room temperature.

Specimens	Tensile Strength (MPa)	Yield Strength (MPa)	Elongation (%)
CNT-AlSi10Mg-1	493.9	300.4	9.8
CNT-AlSi10Mg-2	498.9	311.3	10.6
CNT-AlSi10Mg-3	503	316.8	11.2
Average	498.6	309.5	10.5
AlSi10Mg-1	436.1	261.3	7.4
AlSi10Mg-2	440.1	273.4	7.2
AlSi10Mg-3	441.2	277.3	7.8
Average	439.1	270.7	7.5

## Data Availability

All data included in this study are available upon request by contact with the corresponding author.
